# Sequencing Intractable DNA to Close Microbial Genomes

**DOI:** 10.1371/journal.pone.0041295

**Published:** 2012-07-31

**Authors:** Richard A. Hurt, Steven D. Brown, Mircea Podar, Anthony V. Palumbo, Dwayne A. Elias

**Affiliations:** Biosciences Division, Oak Ridge National Laboratory, Oak Ridge, Tennessee, United States of America; Baylor College of Medicine, United States of America

## Abstract

Advancement in high throughput DNA sequencing technologies has supported a rapid proliferation of microbial genome sequencing projects, providing the genetic blueprint for in-depth studies. Oftentimes, difficult to sequence regions in microbial genomes are ruled “intractable” resulting in a growing number of genomes with sequence gaps deposited in databases. A procedure was developed to sequence such problematic regions in the “non-contiguous finished” *Desulfovibrio desulfuricans* ND132 genome (6 intractable gaps) and the *Desulfovibrio africanus* genome (1 intractable gap). The polynucleotides surrounding each gap formed GC rich secondary structures making the regions refractory to amplification and sequencing. Strand-displacing DNA polymerases used in concert with a novel ramped PCR extension cycle supported amplification and closure of all gap regions in both genomes. The developed procedures support accurate gene annotation, and provide a step-wise method that reduces the effort required for genome finishing.

## Introduction

The emergence of high-throughput next generation DNA sequencing technologies has increased the speed and reduced the cost of genome sequencing [1], [2]. There are many reasons that a closed and finished microbial genome is important, including support for functional genomics, comparative genomics, microbial forensics, and genome organization studies [3], [4]. A finished genome is defined as having less than one nucleotide (nt) error in 10^5^ nt positions with no gaps in the sequence data [5].

Despite this rapid growth in DNA sequencing throughput, there have been few advances in genome finishing, causing a growing disparity in the number of finished genomes and genomes that have gaps in the sequence data deposited in databases [5]. This is in part due to limited advances in sequencing refractory DNA regions, even when complementary or hybrid approaches are used in conjunction with dedicated and directed finishing efforts. Notable advances in computational assembly have reduced the workload involved with acquired sequence data [6], [7], [8], [9]. However, the final stage of genome closure most often requires new data from DNA that is difficult to sequence. Sources of these difficult DNA regions include hairpin structures, elevated %(G + C), homopolymeric stretches, and repeats with numerous approaches and techniques used to overcome some of them [10], [11], [12].

Often with genome finishing, as the number of gaps decreases, the difficulty with closing the remaining gaps increases until no further progress can be made, and the remaining gaps are ruled intractable. This common problem bore enough weight that a new category “non-contiguous finished” was introduced to describe genomes with intractable gaps [5]. Without the ability to sequence such intractable regions, advancements in assembly cannot help. To date, sequencing refractory regions remains dependent on conventional Sanger methods, which are time and cost-intensive [13], [14], [15].

Two “non-contiguous finished”, mercury-methylating, sulfate-reducing bacteria were used in development of the method described herein to finish and close the genomes. The model organism for mercury (Hg) methylation, *Desulfovibrio desulfuricans* strain ND132, was isolated from the Chesapeake Bay, and generates high levels of toxic methylmercury (MeHg) [16], [17]. The ND132 genome was sequenced by the DOE Joint Genome Institute (JGI) to support a comparative genomics approach in identifying genes responsible for Hg methylation [18], [19]. Sequence was generated using Illumina and Titanium 454 DNA technologies [20], [21] and provided 122× coverage and 40× coverage, respectively. Sequencing was followed by extensive standard and “high GC” finishing that produced a genome consisting of one scaffold and six contiguous segments (GenBank CP003220.1). The genome of *D. africanus* was sequenced with similar goals [19], and similar effort resulted in one scaffold and one contiguous segment separated by a single gap (GenBank CP003221.1). A process is presented herein to resolve such recalcitrant regions. The ability to sequence all nucleotides within genomes eliminates gaps that could contain open reading frames, ensuring accurate genome annotation. Faster and less laborious procedures for complete genome closure will allow for more confident comparative and functional genomic studies that can benefit many groups facing similar situations.

## Results

The cause of the sequence gaps in the deposited *D. desulfuricans* ND132 and *D. africanus* genomes were initially described as “hard stops”. During the search for PCR conditions that would support preparation of DNA sequencing templates, an approach for surveying the sequences surrounding each gap was adopted so that commonalities among the recalcitrant regions could be identified. The result from this latter effort suggested a requirement for polymerases and PCR thermal-cycling conditions with tailored properties. Once template amplification conditions were identified, the gaps were sequenced using established amendments to Sanger sequencing. The outcome is a simple process that supports closure of “intractable” sequence gaps caused by self-annealing high %(G + C) DNA to produce finished genomes. Now that the tailored survey approach and amplification conditions have been standardized for Sanger sequencing, this approach can be amended to the standard finishing methods used following the initial next generation sequencing effort.

### Sequence Evaluation

High %(G + C) regions were identified within 100 nt of all gaps that included long tandem iterations of the same nucleotide. Further, nucleotides adjacent to all 6 gaps were high %(G + C) with bases on either side of the gaps complementing to produce secondary (2^o^) structure (Figure S1). The DNA sequence surrounding gaps 1 and 2 exhibited substantial primary (1^o^) and 2^o^ structural similarity. Moreover, alignment searches using 50 nt surrounding gaps 1 and 2 identified a position on the annotated *Desulfovibrio aspoeensis* genome located at a junction between a PhoH family protein on the 5′ side and the formylmethanofuran dehydrogenase subunit E encoded on the 3′ side. DNA folding analysis revealed evidence of 2^o^ structure showing strong thermal stability surrounding all of the gaps in the ND132 genome; except for gap 5, which would not fold at 60°C (Figure S1 and Table S1). Database alignment searches using 100 nt artificial contigs, constructed using 50 nt from either side of each gap, showed that all of the gaps were at or near the junction between two coding regions [22], [23].

### Gap Region Amplification

PCR optimization with *Taq* DNA polymerase included testing various PCR additives and 30 mer primers with no resulting *D. desulfuricans* ND132 amplification products. Initial (30 mer) primers targeted positions several hundred bases from the gaps to prevent amplification failure caused by any potential assembly problems (Table 1). PCR development initiated with *Taq* DNA polymerase used 2 min extensions at 72°C because most amplification products were anticipated to be <2 Kbp. Standard PCR optimization strategies including gradient thermal cycling to determine an optimal annealing temperature, along with variations in Mg^2+^, formamide, dimethylsulfoxide, primer, and template concentrations only produced amplification of gap 4 (Table 2).

**Table 1:** Amplification/Sequencing Primers and Amplicon Electrophoretic Mobility Evaluation.

Primer	Gap	Amplicon Size GAP^a^	Gel Mobility^b^	Estimated GAP Length	Alignment Gap Length^c^
**GAP1F 5′- cgacatcctcaagccctg-3′**	−363	693 bp	700 bp	10 bp	18 bp
**GAP1R 5′- gcaagttcgcggacgtcaac-3′**	+330	“	“	“	“
**GAP2F 5′- ctggccgtcaagaccgac-3′**	−217	508 bp	540 bp	30 bp	30 bp
**GAP2R 5′- tgacgcttcggacgctc-3′**	+291	“	“	“	“
**GAP3F 5′- ctactgcctgcttagcca-3′**	−250	372 bp	375 bp	3 bp	−1 bp
**GAP3P10R 5′-gaattgctccggctttgaaaaatgtaaggc-3′**	+122	“	“	“	“
**GAP4P1F 5′-ggaatgggttcaatcttatcaggttggctcc-3′**	−127	387 bp	400 bp	15 bp^b^	13 bp
**GAP4R 5′-cagccgtgccgtgcctgac-3′**	+260	“			
**GAP5F 5′- gcttcaactcgggcgaac -3′**	−260	449 bp	520 bp	60 bp	ND^c^
**GAP5R 5′- cgagtcgctcacgatggtgtg -3′**	+189	“	“	“	“
**GAP6P2F 5′-cctcaatgtcgcttgatacttgcgtaatcc-3′**	−496	578 bp	620 bp	40 bp	ND
**GAP6P2R 5′-aaggagtaggtttttatgctactacgcccc-3′**	+82	“	“	“	“
**GAP5Fs 5′- gccaacgccctgaacctg -3′**	−129	207 bp	260 bp	60 bp	ND
**GAP5Rs 5′- cacaacgaatgccgctgattc-3′**	+78	“	“		“
**GAP6Fs 5′-ctgcatcgtctaccgaatctg-3′**	−137	241 bp	270 bp	30 bp	ND
**GAP6Rs 5′-cggcgtgtcgggaccgag-3′**	+104	“	“		“

a Amplicon size based on the distance between the 5′ termini of the primer pair excluding any additional length from the gap.

b Electrophoretic mobility rounded to nearest 10 bp based on a combination of Marker III (Roche), Marker V (Roche), and 100 bp ladder (NEB).

c ND means not determined because the artificial contigs did not align in BLAST searches.

**Table 2:** ND132 Template Amplification Summary.

	*D. desulfuricans* ND132 Gaps Resolved		
PCR Method^a^	*Taq* Polymerase	Strand displacing *Pfu* Polymerase	GC-Rich PCR System^b^
Standard Cycling	4	ND	-
Ramped Extension^c^	1,2,3,4	1,2,3,4,5,6	5,6
7′-deaza-2′-dGTP^d^	1,2,3,4,5,6	1,2,3,4,5,6	ND

a Method includes all additives, ions, and annealing and extension temperatures tested.

b Amplification reagent is the GC-Rich PCR System (Roche).

c *Taq* polymerase required 5% or 10% DMSO. *Pfu* polymerase required no additives.

d 7’-deaza-2′-dGTP was used with the ramped extension cycle. When used with *Taq* DNA polymerase, the reaction mixture was supplemented with 1 betaine and 5% DMSO. *Pfu* polymerase required no additives.

A ramped PCR extension cycle (72°C ×1 min and 75°C ×1 min) combined with either 5% or 10% DMSO supported amplification of gaps 1–4 using *Taq* DNA polymerase. However, product generation was inconsistent and invariably produced co-amplified artifacts (Table 2, Figure S2A). The GC-Rich PCR System (Roche) only supported gap 5 and gap 6 amplification using a two-temperature ramped extension segment during thermal-cycling. This was unexpected because gaps 5 and 6 were not amplified with *Taq* DNA polymerase under any conditions. Gap 1 and 2 PCR products reproducibly exhibited distinctive hard stop signatures. There were two artifacts, smaller than the intended products, for each of the six PCRs targeting gaps 1 and 2, likely resulting from polymerase rejection at hard stops (Figure S2B). Amplification using strand-displacing (SD) DNA polymerases eliminated co-amplified artifacts from all gap PCRs. All gap 3 PCRs catalyzed by *Taq* DNA polymerase yielded insufficient product to serve as template in 25 thermal-cycles.

The ramped extension cycle with *Taq* DNA polymerase supported gap 4 amplification without additives. However, all three of the gap 4 PCR products were ∼250 bp smaller than expected, based on the deposited sequence data. Gap 4 forward sequences read into the gap with high quality sequence, while the reverse reaction was correct for the first 200 nt and then diverged to no significant similarity. Also, a 50 nt artificial contiguous DNA sequence constructed using 25 nt from either side of gap 4 did not align with the nearest sequenced relative, *Desulfovibrio aespoeensis.* These findings suggested a potential assembly error with the deposited sequence data surrounding gap 4.

New primers targeting gaps 5 and 6 were designed to generate smaller products (∼600 bp) to determine if the source of amplification difficulty was large fragments with high %(G +C). *Taq* DNA polymerase also failed with the smaller gap 5 and 6 PCRs (Table 2) suggesting that amplicon length in the context of high %(G + C) DNA was not the only source of difficulty. Use of nucleotide analogue 7-deaza-2′-dGTP [12], in concert with 5% DMSO and 1.3 M betaine did support successful amplification of gaps 5 and 6 with *Taq* DNA polymerase [12] (Table 2). In fact, a review of the various amplification procedures used herein reveals that when using primers targeting DNA regions within a few hundred nucleotides of all gaps, all 6 regions could be amplified with either; 1) *Pfu* SD DNA polymerase using either 7-deaza-2′-dGTP or a natural set of nucleotides with a ramped PCR extension cycle or, 2) a ramped PCR extension cycle with *Taq* DNA polymerase, 5% DMSO, 1.3 M betaine and the nucleotide analogue 7-deaza-2′-dGTP (Table 2).

### GAP Sequence Results

**Table 3:** Sequences of the Six *D. desulfuricans* ND132 Gaps.

GAP	Locus^a^	Length	Sequence 5′-3′
1	657659–657675	17	gag ggg gaa cct ctt tc
2	1009606–1009634	29	cttggaagaggttccccctctggactccc
3	1885438	0	
4	2275514 - 2275527	14	aaa agt ttc ccc cc
5	3127972−3128034	63	ccc ttt gag aaa agg gtt ttt cct ccc ctt ccc ccg aac ccc cat ccc ctc ctt tcc cta aac
6	3648109−3648144	36	ctt tgc aaa ggg ttc cct ctc gcc ccc ctt ccc ccc

a Location of gaps in the genome according to updated genome GenBank (CP003220) sequence.

**Figure 1:**
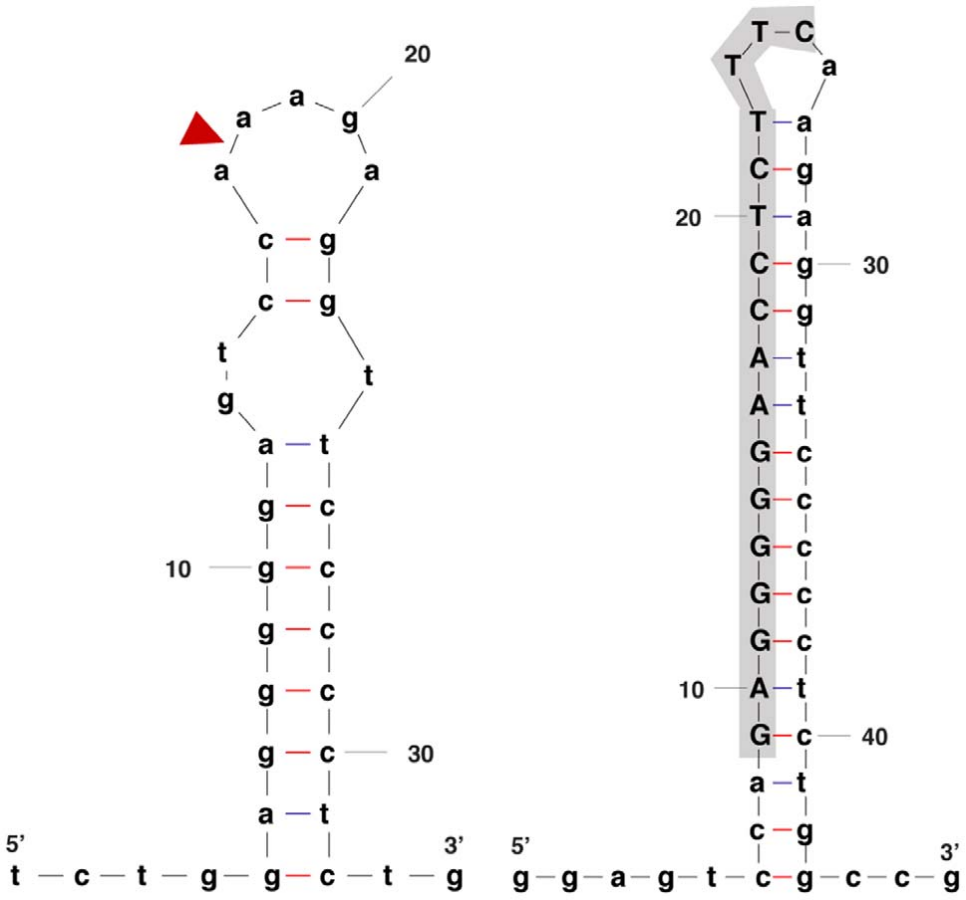
Example of the increase in secondary structure following determination of the gap sequences: Gap 1. Left- secondary structure prediction done using the Mfold web server [29] to aid in primer site selection. Closed triangle shows the location of the gap. Structure was prepared by appending 100 nt at the termini of the 5′ and 3′ contigs surrounding the gap. Folding parameters used 50 mM NaCl, and 2.5 mM MgCl_2_ at 60°C. Excess nucleotides were trimmed from the structure for presentation. Right – secondary structure including the determined gap sequence. The determined gap sequence nucleotides are highlighted in upper case. Folding parameters are as given for the structure prepared prior to gap sequence determination.

The determined length of the gap regions in the ND132 genome ranged from 0 to 63 nt (Table 3). Gap 1 and gap 2 templates were prepared from a cloned source (see methods) and used *Pfu* DNA polymerase with 7-deaza-2′-dGTP. Re-evaluation of the 2^o^ structure following sequence determination showed a considerable increase in thermal stability of the 2^o^ structures for gaps 1 and 2 (Figure 1). The self-complementing sequence was conserved between gaps 1 and 2 with the 5′ complementing region comprising the gap region for gap 1, and the 3′ complementing region comprising the majority of gap 2. The gap 1 forward sequences were weakened by a second hard stop upstream, so gap 1 reverse sequences had higher quality scores (Figure S3). Because gap 2 resided in a different context that lacked a secondary upstream hard stop, the forward sequence data was of good quality.

The gap 4 sequence was determined with extended read-through using standard BigDye® V3.1 reagents and protocols, and revealed a 255 nt assembly error at the 5′ end of the downstream contig with strong 1° and 2° structural similarity with gaps 1 and 2 (Figure S3). With the assembly error corrected, 70 nt surrounding gap 4 aligned with a *D. aespoeensis* genome with 91% identity, bridging the junction between an iron-sulfur type hydrolyase gene at the 5′ side and a succinate dehydrogenase gene on the 3′ side. The misassembled 255 nt shared 79% identity with the *D. aespoeensis* phosphoribosylglycinamide formyltransferase gene. Closure of gap 4 using the determined gap sequence and repair of the assembly error revealed 2^o^ structure that was highly similar to gaps 1 and 2, and may have caused the gap and assembly error. A series of deoxyguanosines within the stem region may have been sufficient to prevent amplification using standard PCR, however, sequencing reactions were successful once gap 4 template was acquired.

The determined gap 3 length was 0 nt compared to the deposited JGI sequence (Table 3). The deposited sequence surrounding gap 3 formed a very strong 2° structure (Figure S4). Gap 3 was flanked by a series of 5, 4, and 3 consecutive guanosines separated by islands of adenines that complemented the opposite side of the gap so that sequencing reactions on both strands were exposed to hard stops, diminishing the sequence trace peak height (Figure S4C). The sequence surrounding gap 3 also had strong 1° and 2° structural similarity with the determined sequence of gap 5. *Pfu* polymerase routinely amplified all of the gap regions, but produced less PCR product with nucleotide mixtures that replace dGTP with 7-deaza-2′-dGTP. The strength (ΔG = −18.13 kcalmol^−1^) and length (28 bp) of the gap 3 2° structure are in agreement with the exceptional amplification and sequencing difficulty.

The small PCR products for gaps 5 and 6 (Table 1) yielded sequences within the gap, however, internal stoppage likely prevented read-through despite the use of 7-deaza-2′-dGTP. Templates prepared with 7-deaza-2′-dGTP yielded long sequencing products that should have spanned the gaps based on the length, but did not. Secondary structure extending back to the primer was observed for gaps 3, 5, and 6. The estimated size of gaps 5 and 6 were 65 and 35 nt, respectively (Table 1). Primers designed closer to gaps 5 and 6 avoided 2^o^ hard stop positions. These smaller products were amplified using *Pfu* DNA polymerase in conjunction with the ramped extension PCR, without the need for additives or 7-deaza-2′-dGTP (Table 2). The surrounding sequence of gap 5 exhibits strong similarity with the gap 3 region.

The gap 5 and 6 sequences were determined using PCR derived template prepared from ND132 genomic DNA using standard nucleotides in conjunction with *Pfu* SD DNA polymerase. Sequencing reactions supplemented with 1 M betaine with an increased extension cycle duration (6 min) supported read through for gap 6, and a 12 bp overlap supported closure of gap 5. Cloned restriction endonuclease fragments yielded clean gap 5 and 6 sequence data, confirming the sequence obtained from the *Pfu* polymerase derived PCR products. The single gap in the *D. africanus* genome was sequenced with the ramped PCR in combination with *Pfu* polymerase and 7-deaza-2′-dGTP. A series of 14 consecutive cytosines present in the deposited *D. africanus* sequence located at the 5′ end of the downstream contig was determined not to exist (Figure S5). Similarly, a series of 17 consecutive cytosines had been reported for gap 6 in prior finishing effort.

## Discussion

The nucleotides surrounding all 6 gaps in the *D. desulfuricans* ND132 genome exhibited extended complementarity with long segments of deoxyguanosine or deoxycytosine causing 2^o^ structure formation. This is likely a common source of difficulty for genome finishing since a survey of 10 additional non-contiguous finished genomes showed that more than half of the gaps had 2° structure (Table 4). Viswanathan *et al.* [24] found that 2^o^ structure caused by miniTn10 transposon insertion resulted in DNA polymerase translating across the base of a stem-loop, resulting in deletion of the majority of the transposon sequence. However, evidence supporting the determined ND132 gap sequences includes closed gap region alignment with *D. aespoeensis* and other bacteria over 100 bps and all 5′ and 3′ terminal nucleotides involved with 2^o^ structure agreed with the contig termini deposited by JGI. Further, none of the gap sites were adjoined to the stem structure base, so there is no reason for any truncation.

**Table 4:** Survey of Additional Non-contiguous Finished Genomes.

Non-contiguous Finished Genome	URLa,b	Total Number of Gaps	Positive for 2° Structure^c^
*Brenneria sp. EniD312*	/bren/	9	5
*Burkholderia cepacia* Bu72	/bcep bu72/	3	3
*Clostridium sp. DL-VIII*	/cspDL/	1	0
*Desulfovibrio africanus*	CP003221	1	1
*Desulfovibrio desulfuricans* ND132	CP003220	6	5
*Desulfovibrio sp. FW1012B*	/dfw101/	2	2
*Frankia sp. EUN1f*	/fran_eun1f/	1	1
*leptonema illini* DSM 21528	/lil21528/	17	5
*Methanofollis liminatans* DSM 4140	/mli4140/	6	3
*Methylocystis sp.* ATCC 49242	/meth4292/	1	1
*Shewanella baltica* OS183	/sbal_183/	4	2
*Thermotogales bacterium* mesG1.Ag.4.2	/tbac/	2	2

a http://genome.ornl.gov/microbial/dfw101/.

b http://www.ncbi.nlm.nih.gov/nuccore/CP003220.

c Secondary structure prior to determination of gap sequences based on location of gap within stem, loop, or adjoined to the stem.

Application of an SD polymerase proved to be a key simplifying factor for routine amplification of refractory regions while the ramped thermal-cycling was initially designed to amplify genes when all standard PCR options failed. Together, all gap regions were routinely amplified with a combination of SD polymerase and ramped extension cycles with all additional cycling parameters constant. The result appears to be a one-size fits all for amplification of difficult templates, and the method should reduce an immense testing burden involving the search for amplification conditions during genome finishing work.

Use of 7-deaza-dGTP supported amplification of all 6 gap regions with either *Taq* or *Pfu* DNA polymerase. Incorporation of 7-deaza-2′-dGTP weakens Watson-Crick base pairing and disturbs base stacking caused by homopolymeric guanidylate making the DNA more amenable to dissociation [25], [26]. Promiscuous nucleotide analogues have been used to insert changes during PCR and make refractory DNA segments amenable to amplification and sequencing [27], but 7-deaza-2′-dGTP is not reported to cause an elevation in mutation rate and so the lack of mismatches in the complementing nucleotides of the gap 2^o^ structures offers confidence in the determined gap sequences. Consistently successful sequencing templates with difficult hard stops were achieved using 7-deaza-2′-dGTP, but gaps 5 and 6 revealed that it alone will not support sequence determination in all cases. Evaluation of the gap 5 sequencing revealed that the sequencing reactions extended into the gap and then self-complemented back to the sequencing primer. This would occur if extension terminated on the downstream side within the gap region by 2^o^ structure and the DNA then folds back on itself forming a hairpin structure. This was why sequencing reactions long enough to have read well onto the downstream contig failed to do so.

Primer proximity also proved to be important since primers closer to the gap regions (∼100 bp away) resulted in more consistent template preparation and improved sequences even though correlations between primer proximity and sequence data quality is weak [28]. Hence, initial primer design should target sites close to the gap with subsequent re-design further away if necessary.

Current high-throughput sequencing methods can leave unreliable sequence at contig termini [15], however, the deposited contig termini in the ND132 genome were correct except for differences at five positions upstream of gap 5. Similarly, the terminus of the downstream side flanking the “intractable” gap in the deposited *D. africanus* genome consisted of 14 cytosines, and like the ND132 gap 6 artifact, was determined not to exist. Digestion and sub-cloning restriction endonuclease fragments provided important support for the ND132 gap 6 DNA sequence.

High %(G + C) 2^o^ structure is the implicated source of difficulty for all tested sequence gaps, although the precise mechanics involved remain poorly understood. The findings presented here make it clear as to why these regions were “intractable” including the 2° structure stem regions containing long regions on complementation such as in gaps 1, 2 and 4 with 10 nt long polypyrimidine tracts while the more difficult gaps 3 and 5 have polypurine tracts 20 and 19 nt long, respectively. Gap 3 was consistently the most difficult region to amplify and this may be due to the tracts of guanosines.

**Figure 2:**
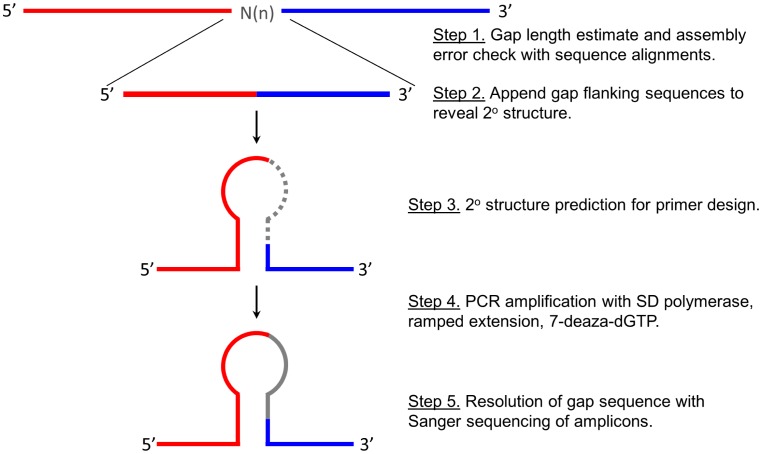
Work Flow Diagram. Top- 100 nt from either side of the gap are appended and used for alignment search to identify and potential problems with assembly causing the gap. N_(n)_ denotes unknown nucleotides (50 or 100 by convention) inserted into the gaps in deposited sequence data The resulting 200 nt artificial contig is subjected to 2° structure analysis. The folding analysis reveals additional 2° structures in the vicinity of the gap that may present added difficulty with amplification and sequencing. Primers are targeted to positions proximal to the gap relative to any additional problems identified in the folding evaluation. Where the gap position is involved with secondary structure, amplification with an SD polymerase and use of a two-step extension cycle (1 min at 72°C followed by 1 min at 75°C) supports amplification. The second segment of the extension cycle can be modified based on the thermal stability identified in the initial folding analysis.

Because of lessons learned herein, a one-size fits-all approach that saves time and effort for the final stages of a finishing effort is presented (Figure 2). The included steps are to first evaluate the available sequence data, removing undetermined nucleotides and append the upstream and downstream contigs to the scaffold. Use 200 nt of the result centered on the gap and run alignment searches to identify assembly errors. Nearest relative alignments can offer an idea on the anticipated size of the gap. Second, run a folding analysis of the surrounding region to rule in or out 2° structure as was the case for all of the intractable gaps herein. A survey of 10 additional non-contiguous finished genomes showed that 57% of 59 gaps involved 2° structure (Table 4). Many of the gaps that did not meet the criteria of being located within a stem structure, within the loop, or adjoined to a stem structure were located in the vicinity of a strong 2° structure. If 2° structure is identified, use a ramped extension cycle in conjunction with a SD polymerase and standard nucleotides. Sequencing reactions done in this assessment used the standard BigDye® Terminator v3.1 reagents except for gaps 5 and 6 where the sequencing reagent was supplemented to 1 M betaine and the extension time was extended from 4 to 6 min for 25 cycles.

Finally, use the gap sequence and surrounding context to run a final alignment search to ensure against truncation has resulted from 2° structure. All 6 of the ND132 gaps were at the junction of two coding regions, and so they may serve as strong transcriptional terminators. The gaps sequenced in this work, along with the cursory survey of other non-contiguous finished genomes show that stable stem-loop structures are a common cause for “intractable” regions, underscoring the importance of structural modeling using the available data surrounding these difficult to sequence regions. Where the type of 2° structure described in this effort is identified, use of ramped extension segments during thermal-cycling in conjunction with SD polymerase are an efficient resource. Moreover, improved sequencing reagents that use a strong SD polymerase should prove equally helpful.

While Sanger sequencing was utilized in the development of this procedure, efforts are underway to optimize both the survey and the amplification conditions to be amenable for next generation sequencing. Automation of this procedure in a high-throughput fashion would be very desirable and would likely be included at the initial stages of finishing. While application of these techniques to improve metagenomic assemblies would be difficult at this time, automation and incorporation into standard next generation finishing protocols could be envisioned for metagenomes as a longer-term goal. However, in the shorter-term, the increased availability of closed, contiguous and truly finished genomes would likely improve contig length and confidence in the longer contig accuracy for community sequencing projects.

## Methods

### Preliminary sequence evaluation

Sequence data surrounding the six gaps in the *D. Desulfuricans* ND132 genome deposited by JGI was examined for high %(G + C) islands and secondary structure. Secondary structure was evaluated by appending 100 nucleotides at the 5′ side to the first 100 nucleotides at the 3′ side of the gaps and submitting the resulting 200 nucleotide contigs to the mFold program [29]. Secondary structure more distant from the gap was noted, and the sequences closer to the gap were re-evaluated by trimming the sequence data to 5 nucleotides on either side of the secondary structural elements located at the gap loci and submitting the resulting artificial contig to mFold. Thermal stabilities for the 6 preliminary mfold structures were determined using SciTools with [Na^+^] = 50 mM [MgCl_2_] = 2.5 mM. The 200 nucleotide artificial contigs (100 nt on either side of the gap loci) were used for alignment searches to identify discontiguous sequence relative related bacterial sequences for identification of potential assembly problems, followed by re-evaluation with the shortened artificial contigs. The thermal stability of the stem regions was evaluated using the DNAanalyzer found in the SciTools environment located on the IDT website using standard PCR parameters of 50 mM Na^+^ and 2.5 mM Mg^++^ to assist with PCR optimization.

### Gap Region Amplification

High molecular weight DNA was extracted from *D. desulfuricans* ND132 cells grown in Walls medium for 3 days as described [30] followed by treatment with DNase free RNaseA (NEB) and adjusted to 1×10^5^ genomes µl^−1^ [30], [31]. Amplification primers (30 mer) targeting the 6 gaps in the *D. desulfuricans* ND132 genome were purchased from Integrated DNA Technologies (Coralville, IO). The initial primer set was designed as 30 nucleotide oligos to insure specificity at high annealing temperatures to support optimization assays. The 6 gaps present in the *D. desulfuricans* ND132 genome are listed in order of appearance on the genome scaffold (Table 2, text). Additional primers were designed to target loci closer to the gaps to bypass additional hard stop loci and generate smaller amplicons for improved sequence data.

PCR optimization assays using *Taq* DNA polymerase were done by varying the annealing temperature between 55°C and 63°C using an Eppendorf® gradient thermal cycler. MgCl_2_ concentration was varied between 1.5 and 3 mM in 0.5 mM increments, and all standard PCR optimization assays were replicated using 5% formamide, 5% DMSO, and 10% DMSO. The listed additive tests were repeated using a ramped PCR extension cycle based on findings from secondary structure evaluations. Ramped extension thermal-cycling used an initial dissociation at 98°C for 3 min followed by 30 cycles of dissociation at 95°C for 30 s, annealing at 59°C for 30 s, extension at 72°C for 1 min and 75°C for an additional minute, with a final extension at 72°C for 7 min. *Pfu* DNA polymerase selected for the properties of strand displacement and high fidelity [32] was expressed from a cloned source, purified using a nickel chelating column (GE Healthcare), and assessed for activity using universal primers targeting the 16S V3/V4 region with *D. desulfuricans* ND132 DNA. 1 unit of *Pfu* DNA polymerase was established as the minimum amount of polymerase that still gave the maximum quantity of PCR product from a 25 thermal-cycle 50 µl reaction in 1 × *Pfu* reaction buffer [20 mM Tris-HCl (pH 8.8), 2 mM MgSO_4_, 10 mM (NH_4_)_2_SO_4_, 10 mM KCl, 0.1 mgml^−1^ BSA, 0.1% Triton X-100]. Following initial testing, all PCRs using *Pfu* DNA polymerase used the ramped extension cycle described above. A GC-Rich PCR System™ (Roche) was used according to manufacturer instructions. *Pfu* DNA polymerase was used to prepare PCR products for cloning to support comparison of sequencing results using plasmid templates with direct sequencing of PCR products.

Illustra™ GFX gel band purification reagents (GE Healthcare) or a QIAquick® Gel Extraction Kit (QIAGEN, Boulder CO). Terminal deoxyadenosine was added using *Taq* DNA polymerase and 1 mM deoxyadenosine 5′ triphosphate in 1×PCR buffer [25 mM Tris-HCl (pH 9.0) 50 mM KCl, 2.5 mM MgCl_2_, 0.1% Triton X-100] at 72°C for 20 min. The resulting tailed *Pfu* amplification products were ligated into pCR2.1 (Invitrogen) using a TOPO™ TA cloning kit (Invitrogen®) and plasmids were grown in *Escherichia coli* TOP 10 cells (Invitrogen®). Because of the difficulty with gap region amplification, recombinant screening required preparation of plamids by alkaline lysis followed by purification with organic extraction. Plasmid screening was done with PCR primers specific for pCR2.1, using the ramped thermal-cycling process described previously.

Once amplification conditions were established for all 6 gap regions sequencing template was prepared using *Pfu* DNA polymerase. In addition to PCR products prepared using a standard mixture of deoxyribonucleotide triphosphates (dNTPs), templates were prepared using 7-deaza-2′- deoxyguanosine-5′-triphosphate (USB, Cleveland OH) or CleanAmp™ 7-deaza-2′- deoxyguanosine-5′-triphosphate (TriLink Technologies San Diego CA) to improve the quality of sequencing results [33]. Gap 3 template production using *Pfu* DNA polymerase was consistemly weak, and was therefore prepared using *Taq* DNA polymerase in conjunction with 1 M betaine and 5% DMSO.

### Gap Sequencing

BigDye™ V3.1 (Applied Biosystems) was used for all sequencing reactions. Tested amendments included 5% DMSO and 1 M betaine. Sequences for gaps 1, 2, 3, and 4 were obtained using a combination of 5% DMSO and 1 M betaine under standard cycling conditions that included an initial hold at 96°C ×1 min followed by [96°C ×10 s, 50°C ×10 s, and 60°C ×4 min] over 25 cycles. Gaps 5 and 6 were pre-incubated at 98°C for 3 min in a solution containing primer and sufficient betaine to give a final concentration of 1 M following the addition of sequencing reagent. BigDye™ V3.1 sequencing reagent added according to manufacturer reccomendations and thermal-cycled as described above with a modification of the extension time to 6 min.

### Separation of Hairpin Structures

Gap 5 and gap 6 were amplified using *Pfu* DNA polymerase and evaluated with a battery of restriction endonucleases. Based on a digestion assessment, restriction endonuclease *Hae*III and *Msp*I digests were cloned into pCR2.1 (Invitrogen) using TOPO TA cloning reagents. Terminal deoxyadenine was added by incubating the *Hae*III digestion products with 1 U *Taq* DNA polymerase and 1 mM dATP in 1 × PCR buffer [50 mM KCl, 25 mM Tris (pH 9.0), 0.1% Triton X-100] supplemented with 2.5 mM MgCl_2_. *Msp*I digestion products have recessed 3′ termini requiring extension with terminal dexoyadenine addition using dATP, dCTP and dGTP at 330 µM each. Because of difficulty with amplification through the hard stop loci, clone screening required plasmid preparation by alkaline lysis. Constructs containing inserts were sequenced using plasmid complementing primers TAF 5′-GCC GCC AGT GTG CTG GAA TT-3′ and TAR 5′-TAG ATG CAT GCT CGA GCG GC-3′.

### Data Access

The original non-contiguous finished genomes for *D. africanus* (CM001160.1) and *D. desulfuricans* ND132 (CM001077.1) were deposited in GenBank and can be found at (http://www.ncbi.nlm.nih.gov/nuccore/CM001160.1?report=genbank) and (http://www.ncbi.nlm.nih.gov/nuccore/CM001077.1?report=genbank), respectively. The closed genomes as a result of this work for *D. africanus* (CP003221) and *D. desulfuricans* ND132 (CP003220) can be found at (http://www.ncbi.nlm.nih.gov/nuccore/CP003221) and (http://www.ncbi.nlm.nih.gov/nuccore/CP003220), respectively.

## Supporting Information

Figure S1 **Pre- gap determination 2° structure evaluation.** Mfold 2° structures for gaps 1, 2, 5, and 6 (left to right) prepared prior to determination of gap sequences are shown. Mfold structures were generated by appending the 5′ and 3′ proximal nucleotides flanking each gap so that the preliminary structures shown lack the gap nucleotides. Blue arrows show the site of the gap for each structure. DNA was folded using standard PCR conditions (50 mM Na^+^ and 2.5 mM Mg^2+^). All folds were performed using 60°C except gap 5 (37°C). Note mismatches in the gap 5 stem structure (black arrows).
(DOC)

Figure S2 **Amplification of** ***D. desulfuricans*** **ND132 sequencing templates.** (A) *Taq* DNA polymerase (10% DMSO) PCR products from gap 1–6 using a ramped extension cycle consisting of 1 min at 72°C followed by 1 min at 75°C over 30 thermal-cycles. For each gap, 20 µl from each of 3 different PCRs is electrophoresed. Gap 3 products show the typical low yield for each of the 30 mer primed PCRs. Lane M; Marker III™ (Roche). (B) Expanded view of artifacts from gaps 1 and 2. For gap 1 and gap 2 sufficient product is generated so that 2 artifacts are visible for each of the 3 PCRs targeting both gap regions. P- product; A_1_ and A_2_ indicate the position of artifacts that are generated for all 6 PCRs using *Taq* DNA polymerase for gaps 1 and 2. Gap 4 amplifies without difficulty and does not produce the 2 artifacts even though the 2°C structure shares strong similarity with gaps 1 and 2 (see figure 3).
(DOC)

Figure S3 **Secondary Structure Assessment of Gap 1, 2, and 4.** Mfold structure for Gaps 1, 2, and 4 showing 1° and 2° structural similarity determined by placing the determined gap sequence within 21 nucleotides of context on both the 5′ and 3′ sides of the determined sequence. Polypurine tracts 10 nt long occur in the 5′ stem complement of the gap 1 and 2 structures that complement polypyrimidine tracts in the 3′ side of the stems. Gap 4 also has a 10 nt polypurine tract in the 5′ stem sequence that contains a stretch of 6 consecutive guanosines. All gap sequences determined using the current process are shown in upper case. Mfold parameters used default settings at (60°C; [Na^+^] = 50 mM, [Mg^++^] = 2.5 mM). Gap sequence data quality: gap 1 Q = 94.4%, gap 2 Q = 94.5% gap 4 Q = 73.0% as called by Sequencher® Version 4.9).
(DOC)

Figure S4 **Sources of gap sequencing difficulty.** (A) Gap 3 1° and 2° structure. The nucleotides (A-T) surrounding the site of the non-extant gap in the sequence data are shown in upper case on the folded structure. The sequence is trimmed to the limit of the stem region complementarity. A 20 nt polypurine tract comprises the majority of the 5′ complement of the stem structure (nt 3–23). Folding parameters include 60°C, 50 mM Na^+^, and 2.5 mM Mg^++^ to match conditions used for primer annealing in gap region amplification. (B) The gap 3 sequence trace showing nucleotide positions (black arrows) where the determined sequence diverges from the sequence determined by prior next-generation sequencing and finishing work (Q = 93.0% by Sequencher™ 4.9). The sequence trace shows the typical effect of a hard stop locus with elevated peaks between nucleotide positions 250 to 270 followed by diminished peak height. The elevated peaks of the polypurine tract complement the downstream polypyrimidine tract (nucleotides 285–305). Sequence changes marked −C and + T indicate nucleotides absent or inserted relative to prior data giving a net gap length of 0 nt. (C) Model for self-priming effect observed for gaps 3, 5, and 6. (1) Polymerase extension product having a 3′ terminal segment (light blue) that complements a series of nucleotides upstream on the extension product and is terminated by polymerase halting at the site of a hard-stop (light blue arrow) instead of by incorporation of a labeled terminator during an extension cycle. (2) Fold-back of the complementing segment of the sequencing reaction extension product forms a hairpin structure. (3) The self-annealed 3′ terminus of the hairpin structure primes extension during the subsequent annealing segment of the cycle sequencing program. The mfold structure shown below is a representative fold of a sequencing product for gap 5 derived from template containing 7′-deaza-2′-dGTP instead of dGTP.
(DOC)

Figure S5 ***Desulfovibrio africanus*** **gap structure.** (A) Folding structure prepared using 200 nt centered on the gap. Arrow indicates the location of the gap prior to determination of the gap sequence. Structure was determined using mfold through SciTools located on the Integrated DNA Technologies web site. (B) Determined structure. Gap nucleotides were determined using template prepared with 7-deaza-2′- deoxyguanosine-5′-triphosphate (TriLink Technologies San Diego CA) in conjunction with *Pfu* DNA polymerase and sequenced using standard BigDye™ procedure (Q = 73.9%, forward and reverse sequences, by Sequencher™ 4.9). Determined gap nucleotides are shown in uppercase. The structure was determined using the Mfold website. Thermodynamic data; ΔG = −6.81 kcal/mol at 60°C; T_m_ = 87.8°C; assuming a 2 state model. Ionic conditions: [Na^+^] = 0.05 M, [Mg^++^] = 0.0025 M.
(DOC)

Table S1 (DOC)
