# Supplementary figures and images for: Sequencing Intractable DNA to Close Microbial Genomes
Source: PLoS One. 2012 Jul 31;7(7):e41295. doi: 10.1371/journal.pone.0041295 (PMC3409199; doi:10.1371/journal.pone.0041295)

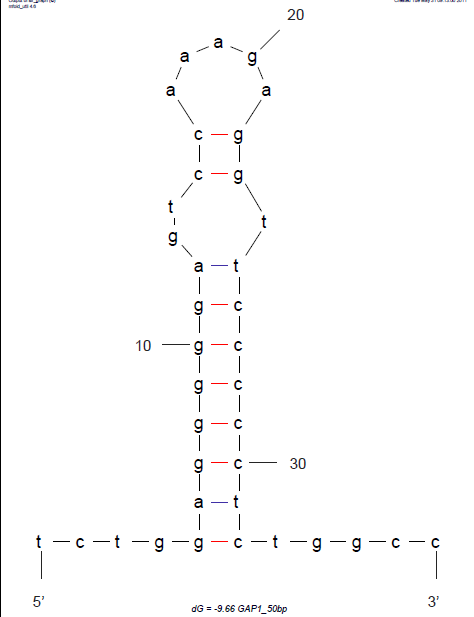

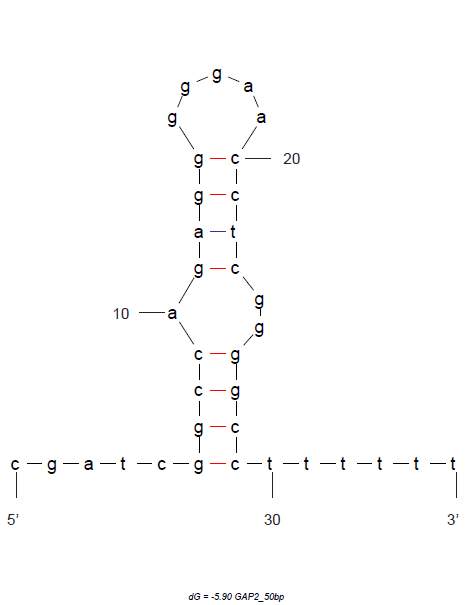

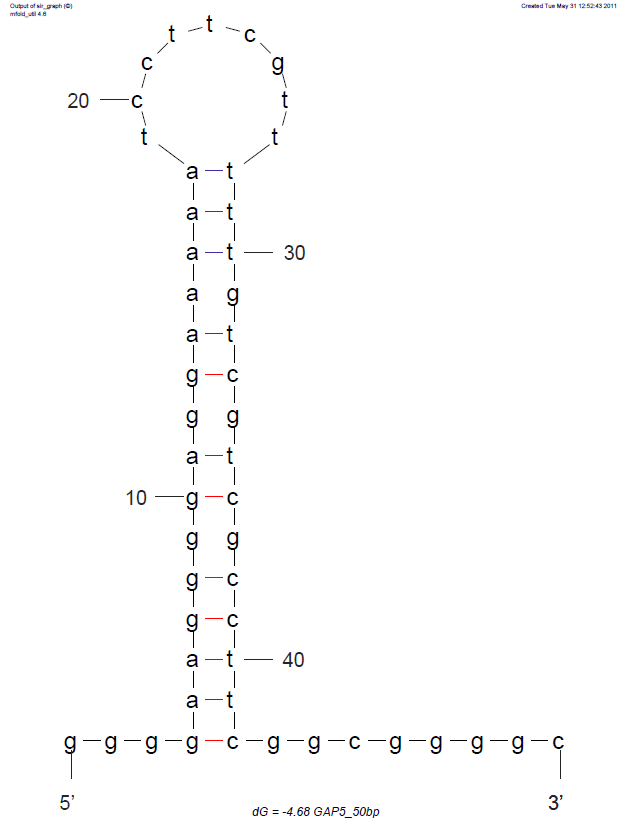

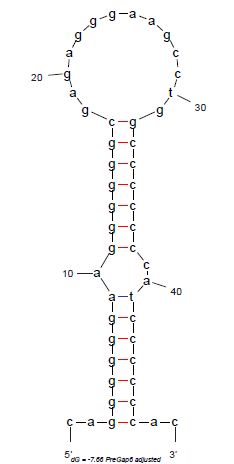

Supplement: Figure S1 — Pre- gap determination 2° structure evaluation. Mfold 2° structures for gaps 1, 2, 5, and 6 (left to right) prepared prior to determination of gap sequences are shown. Mfold structures were generated by appending the 5′ and 3′ proximal nucleotides flanking each gap so that the preliminary structures shown lack the gap nucleotides. Blue arrows show the site of the gap for each structure. DNA was folded using standard PCR conditions (50 mM Na+ and 2.5 mM Mg2+). All folds were performed using 60°C except gap 5 (37°C). Note mismatches in the gap 5 stem structure (black arrows). (DOC) [file pone.0041295.s001.doc]

**Gap 1 Gap 2 Gap 3 Gap 4 Gap 5 Gap 6**

**M A B C A B C A B C A B C A B C A B C**

**M G1 G2**

**(A)**

**(B)**

Kbp

21.2

5.1-3.5

2.1

1.6

1.4

.95-.83


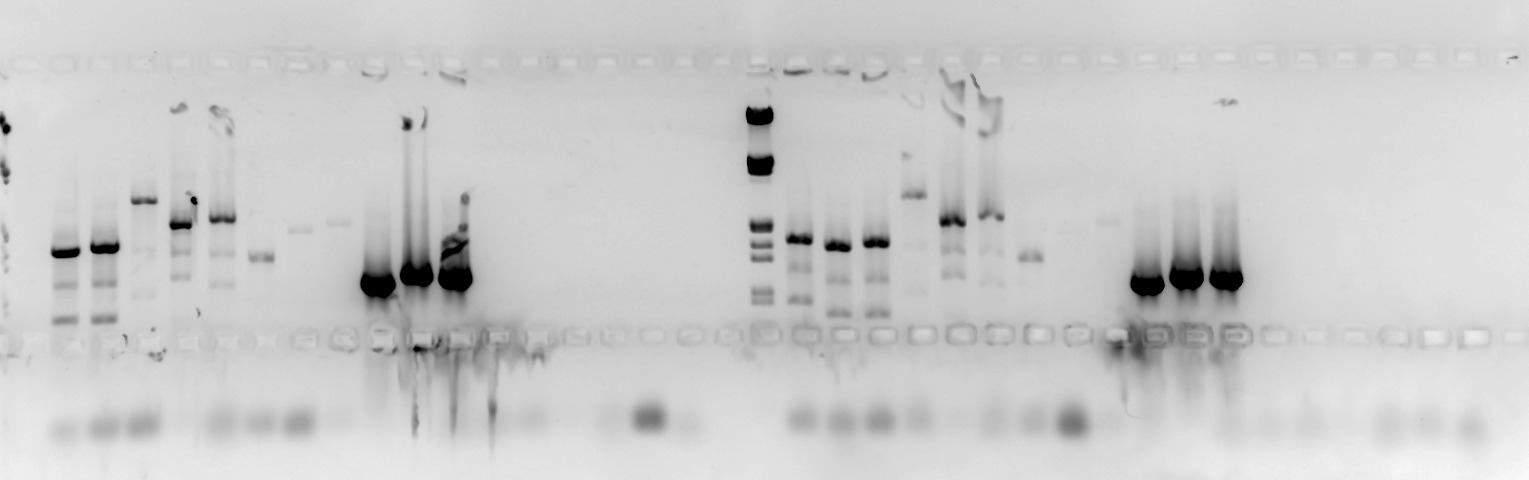


**P**

A1

A2


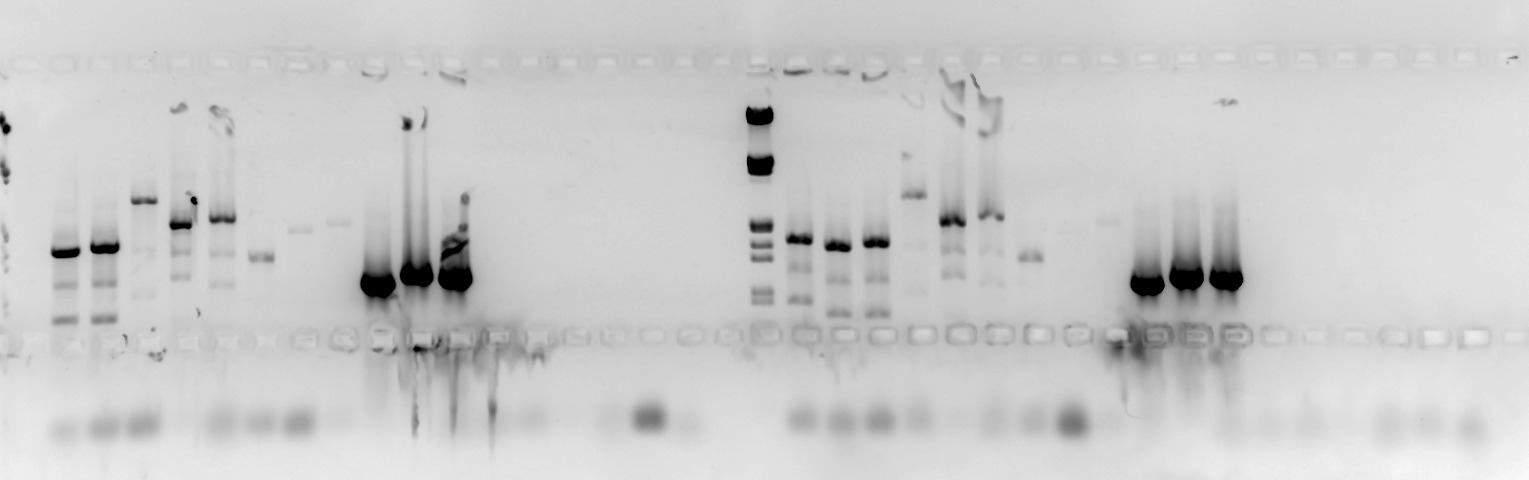


**P**

A1

A2


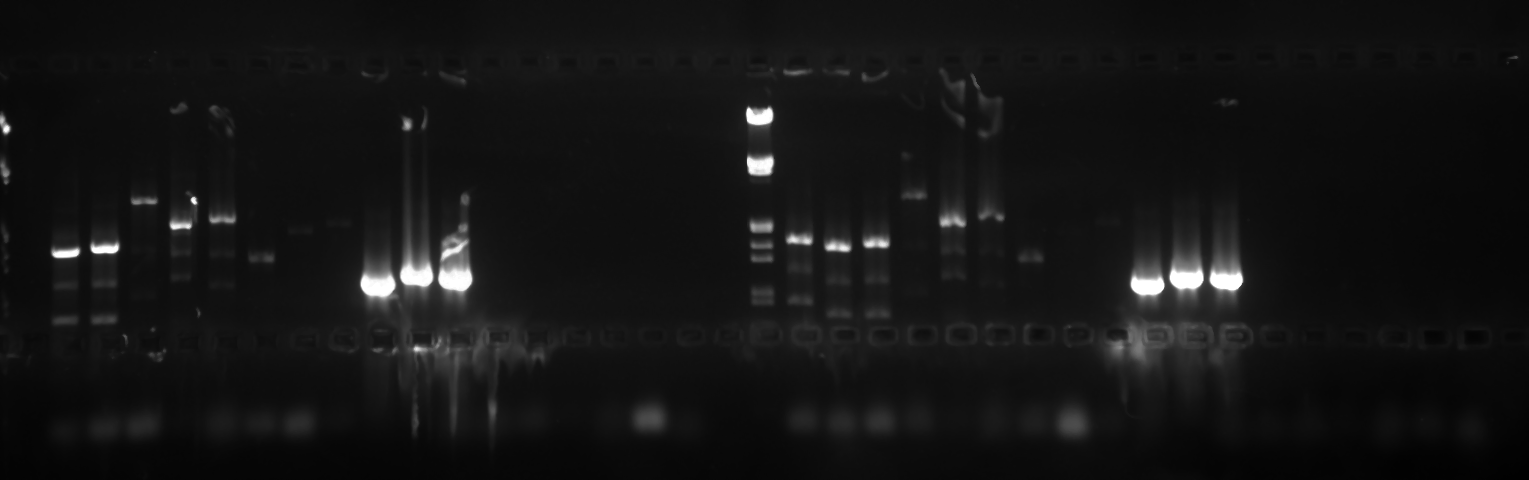

Supplement: Figure S2 — Amplification of D. desulfuricans ND132 sequencing templates. (A) Taq DNA polymerase (10% DMSO) PCR products from gap 1–6 using a ramped extension cycle consisting of 1 min at 72°C followed by 1 min at 75°C over 30 thermal-cycles. For each gap, 20 µl from each of 3 different PCRs is electrophoresed. Gap 3 products show the typical low yield for each of the 30 mer primed PCRs. Lane M; Marker III™ (Roche). (B) Expanded view of artifacts from gaps 1 and 2. For gap 1 and gap 2 sufficient product is generated so that 2 artifacts are visible for each of the 3 PCRs targeting both gap regions. P- product; A1 and A2 indicate the position of artifacts that are generated for all 6 PCRs using Taq DNA polymerase for gaps 1 and 2. Gap 4 amplifies without difficulty and does not produce the 2 artifacts even though the 2°C structure shares strong similarity with gaps 1 and 2 (see figure 3). (DOC) [file pone.0041295.s002.doc]

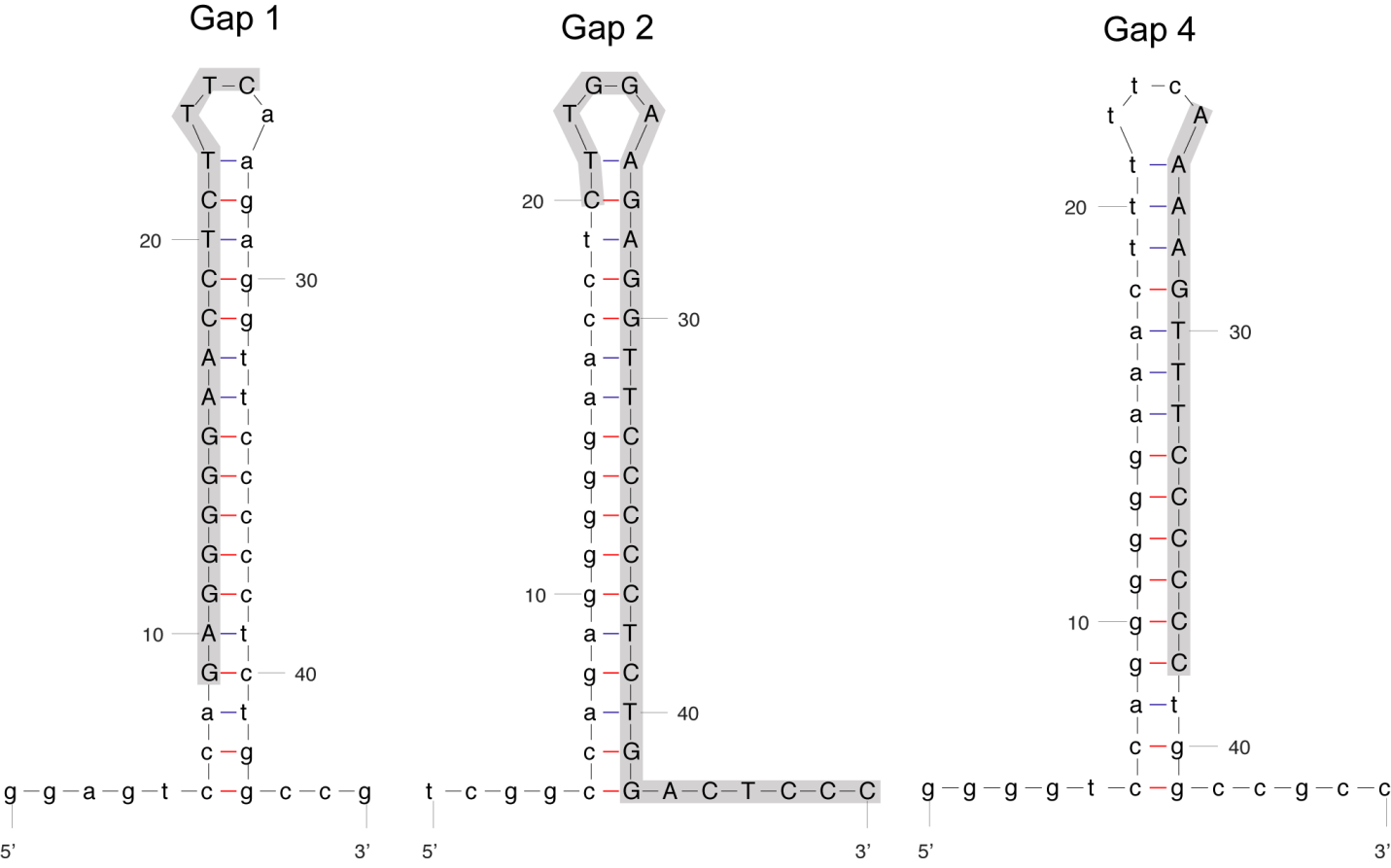

Supplement: Figure S3 — Secondary Structure Assessment of Gap 1, 2, and 4. Mfold structure for Gaps 1, 2, and 4 showing 1° and 2° structural similarity determined by placing the determined gap sequence within 21 nucleotides of context on both the 5′ and 3′ sides of the determined sequence. Polypurine tracts 10 nt long occur in the 5′ stem complement of the gap 1 and 2 structures that complement polypyrimidine tracts in the 3′ side of the stems. Gap 4 also has a 10 nt polypurine tract in the 5′ stem sequence that contains a stretch of 6 consecutive guanosines. All gap sequences determined using the current process are shown in upper case. Mfold parameters used default settings at (60°C; [Na+] = 50 mM, [Mg++] = 2.5 mM). Gap sequence data quality: gap 1 Q = 94.4%, gap 2 Q = 94.5% gap 4 Q = 73.0% as called by Sequencher® Version 4.9). (DOC) [file pone.0041295.s003.doc]

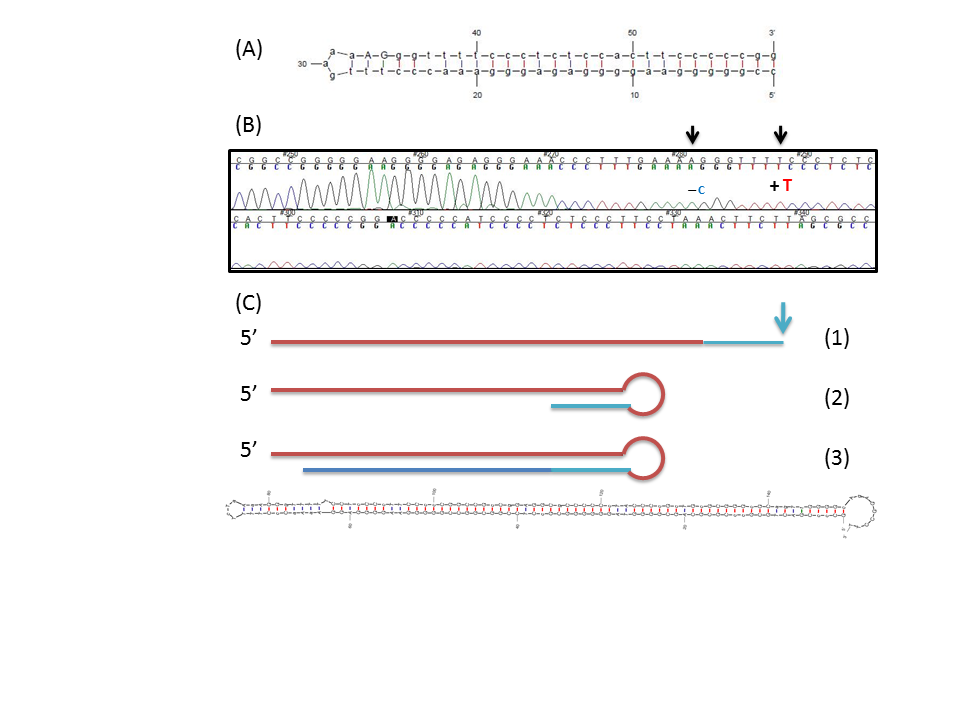

Supplement: Figure S4 — Sources of gap sequencing difficulty. (A) Gap 3 1° and 2° structure. The nucleotides (A-T) surrounding the site of the non-extant gap in the sequence data are shown in upper case on the folded structure. The sequence is trimmed to the limit of the stem region complementarity. A 20 nt polypurine tract comprises the majority of the 5′ complement of the stem structure (nt 3–23). Folding parameters include 60°C, 50 mM Na+, and 2.5 mM Mg++ to match conditions used for primer annealing in gap region amplification. (B) The gap 3 sequence trace showing nucleotide positions (black arrows) where the determined sequence diverges from the sequence determined by prior next-generation sequencing and finishing work (Q = 93.0% by Sequencher™ 4.9). The sequence trace shows the typical effect of a hard stop locus with elevated peaks between nucleotide positions 250 to 270 followed by diminished peak height. The elevated peaks of the polypurine tract complement the downstream polypyrimidine tract (nucleotides 285–305). Sequence changes marked −C and + T indicate nucleotides absent or inserted relative to prior data giving a net gap length of 0 nt. (C) Model for self-priming effect observed for gaps 3, 5, and 6. (1) Polymerase extension product having a 3′ terminal segment (light blue) that complements a series of nucleotides upstream on the extension product and is terminated by polymerase halting at the site of a hard-stop (light blue arrow) instead of by incorporation of a labeled terminator during an extension cycle. (2) Fold-back of the complementing segment of the sequencing reaction extension product forms a hairpin structure. (3) The self-annealed 3′ terminus of the hairpin structure primes extension during the subsequent annealing segment of the cycle sequencing program. The mfold structure shown below is a representative fold of a sequencing product for gap 5 derived from template containing 7′-deaza-2′-dGTP instead of dGTP. (DOC) [file pone.0041295.s004.doc]

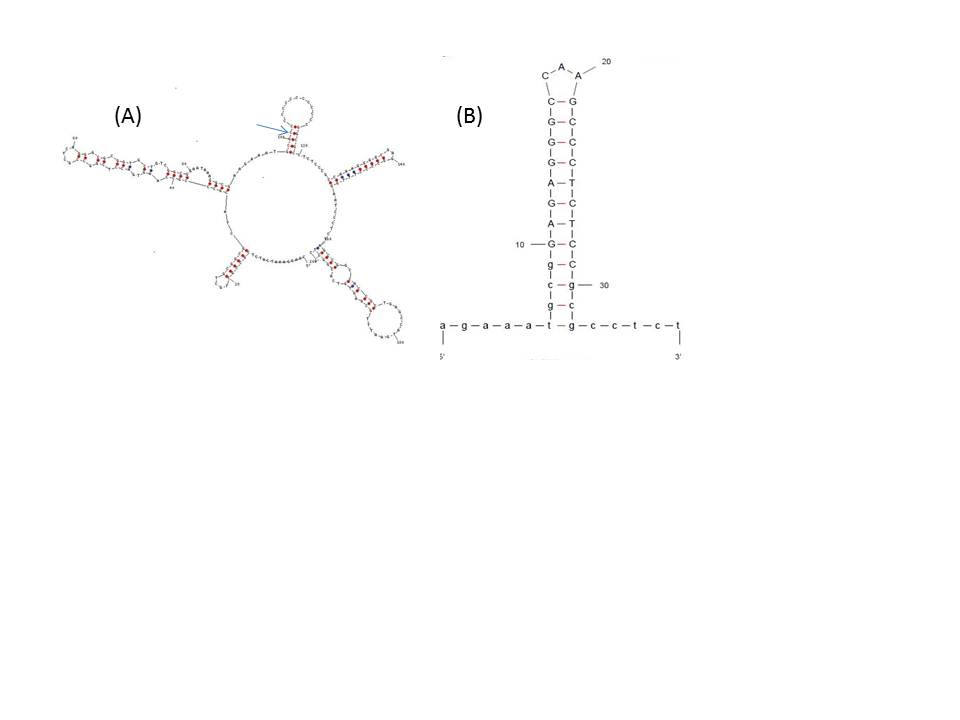

Supplement: Figure S5 — Desulfovibrio africanus gap structure. (A) Folding structure prepared using 200 nt centered on the gap. Arrow indicates the location of the gap prior to determination of the gap sequence. Structure was determined using mfold through SciTools located on the Integrated DNA Technologies web site. (B) Determined structure. Gap nucleotides were determined using template prepared with 7-deaza-2′- deoxyguanosine-5′-triphosphate (TriLink Technologies San Diego CA) in conjunction with Pfu DNA polymerase and sequenced using standard BigDye™ procedure (Q = 73.9%, forward and reverse sequences, by Sequencher™ 4.9). Determined gap nucleotides are shown in uppercase. The structure was determined using the Mfold website. Thermodynamic data; ΔG = −6.81 kcal/mol at 60°C; Tm = 87.8°C; assuming a 2 state model. Ionic conditions: [Na+] = 0.05 M, [Mg++] = 0.0025 M. (DOC) [file pone.0041295.s005.doc]
