# Supplementary material for: Sequencing Intractable DNA to Close Microbial Genomes
Source: PLoS One. 2012 Jul 31;7(7):e41295. doi: 10.1371/journal.pone.0041295 (PMC3409199; doi:10.1371/journal.pone.0041295)
Supplement: Table S1 — (DOC) [file pone.0041295.s006.doc]

**Table S1. Pre-gap Determination vs Completed Secondary Structure Data**

| **GAP/ Locus** | **Stem duplex Length/**  **%(G +C)** | **Gap^−^ 2° structure**  **ΔG_1_ at 60 °C kcal/mol** | **T*_d_* _(adjusted)_^a^** | **Completed Stem duplex Length/ %(G +C)** | **ΔG_2_ kcal/mol** | **T*_d_* mFold/**  **T*_d_* basic for Stem region** |
| --- | --- | --- | --- | --- | --- | --- |
| (1) 351931 | 8 nt/75% | ΔG = -2.50 | 77.6 °C | 17nt/65% | -10.26 | 86.7°C/ 62.5 °C |
| (2) 1227785 | 4 nt/100%  &^b^ 4 nt/75% | ΔG = -1.13 | 65.3 °C | 17nt/65% | -10.93 | 88.0°C/ 65.2°C |
| (3) 1617910 | 28 nt/64% | ΔG = -18.13 | 92.2 °C | 28 nt/61% | -18.13 | 92.2°C/ 75.3°C |
| (4) 2470666 | 7 nt/71% | ΔG = -2.50 | 77.6 °C | 16 nt/56% | -8.74 | 84.1°C/ 58.7°C |
| (5) 2990790 | 15 nt/47% | ΔG^c^ = -4.68 | 53.9 °C | 26 nt/54% | -18.45 | 91.7 °C/ 71.8°C |
| (6) 3858934 | 7 nt/86% &  7 nt/100% | ΔG^b^ = -7.66 | 83.4 °C | 20 nt/75% | -13.61 | 93.1 °C/ 72.4°C |

^a^ Thermal stability adjusted for only 2.5 mM MgCl_2_ and 50 mM Na^+^ mFold result.

^b^Where thermal stability is from two stem regions separated by a bulge, individual duplexed T*_d_* is given.

All calculations were done using 50 mM Na^+^; 2.5 mM Mg^++^.

^c^ΔG at 37 °C because no folding product at 60°C.
